# Reporting of uterine fibroids on ultrasound examinations: an illustrated report template focused on surgical planning

**DOI:** 10.1590/0100-3984.2022.0048

**Published:** 2023

**Authors:** Michel Santos Palheta, Francisco das Chagas Medeiros, Ana Roberta Gomes Severiano

**Affiliations:** 1 Universidade Federal do Ceará (UFC), Fortaleza, CE, Brazil; 2 Centro Universitário Inta (Uninta), Sobral, CE, Brazil

**Keywords:** Ultrasonography, Leiomyoma, Myoma, Uterine myomectomy, Metrorrhagia, Infertility, Ultrassonografia, Leiomioma, Mioma, Miomectomia uterina, Metrorragia, Infertilidade

## Abstract

Uterine fibroids are the most common benign gynecologic tumors in women of reproductive age, and ultrasound is the first-line imaging modality for their diagnosis and characterization. The International Federation of Gynecology and Obstetrics developed a system for describing and classifying uterine fibroids uniformly and consistently. An accurate description of fibroids in the ultrasound report is essential for planning surgical treatment and preventing complications. In this article, we review the ultrasound findings of fibroids, detailing the main points to be reported for preoperative evaluation. In addition, we propose a structured, illustrated report template to describe fibroids, based on the critical points for surgical planning.

## Abstract (pt)

Os miomas uterinos são os tumores ginecológicos benignos mais comuns em mulheres em idade reprodutiva, sendo a ultrassonografia a modalidade de imagem de primeira linha para seu diagnóstico e caracterização. A Federação Internacional de Ginecologia e Obstetrícia desenvolveu um sistema para descrever e classificar os miomas uterinos de forma uniforme e consistente. Uma descrição precisa dos miomas no laudo ultrassonográfico é essencial para o planejamento do tratamento cirúrgico e prevenção de complicações. Neste artigo, revisamos os achados ultrassonográficos de miomas, detalhando os principais pontos a serem relatados para avaliação pré-operatória. Além disso, propomos um modelo de relatório estruturado e ilustrado para descrição de miomas, com base nos pontos críticos para o planejamento cirúrgico.

## INTRODUCTION

Uterine fibroids are the most common benign gynecological tumors in women of reproductive age^(1,2)^. Most women with fibroids are asymptomatic, and nearly a third of patients have significant symptoms such as dysmenorrhea, menorrhagia, abnormal uterine bleeding, secondary anemia, pelvic pain, and infertility^(1,2)^. The treatment of patients with uterine fibroids should be individualized on the basis of the symptoms, patient age, patient desire to preserve fertility or the uterus, and the characteristics of the nodules (e.g., size and location), as well as the availability of therapy and the experience of the attending physician^(2,3)^. In this context, ultrasound is considered the initial test of choice for the diagnosis of fibroids in symptomatic patients, mainly due to its broad availability, ease of use, cost-effectiveness, high sensitivity, and high specificity^(4,5)^. The examination should be performed by specially trained physicians, with the aim of accurately identifying and describing all fibroids^(4,5)^. Other aspects that are crucial in the choice of treatment—the size and location of fibroids; the presence and size of the submucosal component; penetration of the myometrial component; proximity to the uterine serosa; relationship with and proximity to the endometrial cavity; vascular supply; and coexistence of adenomyosis or deep endometriosis—are easily determined and can be characterized by using transvaginal ultrasound^(5,6,7)^.

In 2011, the Fédération Internationale de Gynécologie et d’Obstétrique (FIGO) published a classification system for categorizing the location of uterine fibroids^(8)^. The Morphological Uterus Sonographic Assessment (MUSA) group subsequently ratified the FIGO classification, adopting it to describe the location of fibroids^(9,10)^. Although the FIGO classification system has provided gynecologists with a well-standardized framework for describing and characterizing uterine fibroids, significant variability has been observed across ultrasound reports in terms of the FIGO classification^(11)^. Errors in the classification and description of fibroids in imaging reports can lead to inappropriate surgical planning^(7,11)^. However, it is well known that the accuracy of ultrasound depends on the skill of the performing physician and the quality of the description in the ultrasound report^(12,13)^. Therefore, the use of structured reports, divided into ordered sections and with standardized language, could improve the communication of the results of ultrasound examinations and the confidence of the gynecologist in those results^(14)^.

In the present study, we illustrate the main findings to be reported in an ultrasound report of fibroids. We also propose a structured template for transvaginal ultrasound reports, designed to facilitate the preoperative evaluation of patients with uterine fibroids.

## CLASSIFICATION OF FIBROIDS

**Figure 1:**
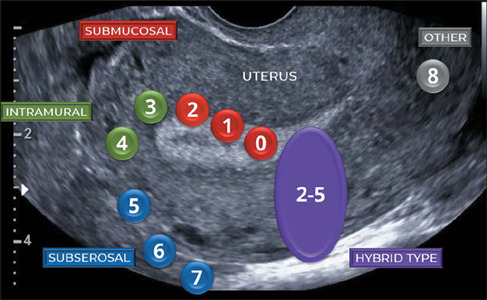
FIGO classification of fibroids: 0 = pedunculated intracavitary fibroid; 1 = submucosal fibroid that is < 50% intramural; 2 = submucosal fibroid that is ≥ 50% intramural; 3 = fibroid that is 100% intramural but in contact with the endometrium; 4 = intramural fibroid; 5 = subserosal fibroid that is ≥ 50% intramural; 6 = subserosal fibroid that is < 50% intramural; 7 = sub-serosal pedunculated fibroid; 8 = other (e.g., cervical and parasitic) fibroids; and 2-5 = hybrid fibroid that is < 50% submucosal and < 50% subserosal.

Traditionally, the classification of fibroids is based on their location in relation to two anatomical planes^(15)^: the endometrium and the uterine serosa. Thus, uterine fibroids are classified as submucosal, intramural, or sub-serosal^(16)^. With advances in diagnostic modalities, the need arose for a detailed, universally accepted classification system as a guide for choosing the most appropriate treatment^(17)^. Therefore, in 2011, the FIGO classification system for causes of abnormal uterine bleeding was developed^(17,18)^. Currently, the FIGO classification includes a total of nine types of fibroids^(8)^—types 0 through 8—as presented in Table 1 and Figure 1.

**Table 1:** FIGO classification of fibroids.

Localization	Type	Description
Submucosal	0	Pedunculated intracavitary fibroid (i.e., submucosal fibroid without intramural extension)
	1	Submucosal fibroid with intramural extension < 50%
	2	Submucosal fibroid with intramural extension > 50%
Intramural	3	Intramural fibroid in contact with the endometrium but not extending into the uterine cavity or serous surface
	4	Intramural fibroid without contact with the endome-trium and without extension into the uterine cavity or serous surface
Subserosal	5	Subserosal fibroid with intramural extension > 50% and < 50% subserosal
	6	Subserosal fibroid with intramural extension < 50% and > 50% subserosal
	7	Subserosal pedunculated fibroid
Other	8	Other types of fibroids (e.g., cervical, broad ligament, and parasitic fibroids)
Hybrid type	2-5	Hybrid classification used when a fibroid extends from the endometrial cavity to the serosa, composed of two numbers, separated by a hyphen, the first characterizing the relationship between the fibroid and the endometrium and the second characterizing its relationship with the serosa

The FIGO classification system was revised in 2018^(19)^. The revised version suggests that an estimate of the total uterine volume should be provided in the ultrasound report, as should the estimated total number of fibroids. In addition, the report should include the estimated volumes of up to four fibroids and their locations, described as anterior, posterior, right, left, or fundus. Furthermore, the relationship between the endometrium and fibroids should be recorded in accordance with the FIGO classification system^(19)^.

## ULTRASOUND DIAGNOSIS OF UTERINE FIBROIDS

**Figure 2:**
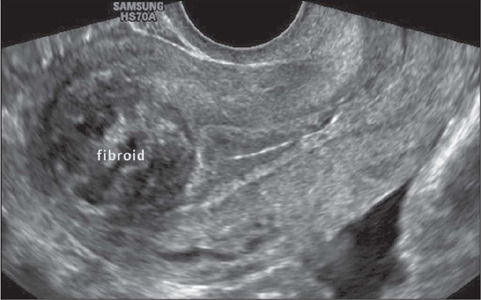
Transvaginal ultrasound image showing a submucosal uterine fibroid.

**Figure 3:**
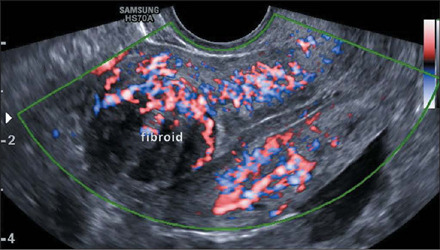
Transvaginal color Doppler ultrasound image showing a submucosal fibroid with circumferential vascularity.

On ultrasound, a uterine fibroid is classically characterized as a solid, round, well-defined, hypoechoic, heterogeneous lesion within the myometrium, often showing acoustic shadowing at the edge of the lesion, with or without internal fan-shaped shadowing (Figure 2). On color Doppler (Figure 3), the circumferential flow around the lesion is often visible^(20)^. In addition, Fleischer et al.^(21)^ successfully used three-dimensional (3D) color Doppler to demonstrate that hypervascular fibroids show a greater reduction in size after uterine artery embolization than do isovascular and hypovascular fibroids. Those authors also found that, after the procedure, standard ultrasound showed decreased uterine size and echogenicity and color Doppler imaging showed a marked decrease in blood flow to the leiomyoma.

The 2015 MUSA consensus suggested using a systematic approach to assessing and reporting ultrasound findings of the myometrium and associated fibroids^(20,22)^. The relevant parameters are presented in Table 2.

**Table 2:** The MUSA consensus.

Parameter	Criteria
Uterus	Measurement of length, anteroposterior diameter, transverse diameter, and volume
Serosal contour	Regular or lobulated
Myometrial walls	Symmetrical or asymmetrical
Myometrial echogenicity	Homogeneous or heterogeneous
Myometrial lesions	Margins — Well-defined or ill-defined
	Number of lesions
	Location — Anterior , posterior, fundal, right/ left lateral, or global
	Type — According to the FIGO classification
	Size — Three perpendicular diameters
	Outer lesion-free margin — Distance from the serosal surface
	Inner lesion-free margin — Distance from the endometrial surface
	Echogenicity — Hypoechoic, isoechoic, or hy-perechoic

## KEY POINTS FOR THE SURGICAL TREATMENT OF FIBROIDS

Decisions regarding the treatment of fibroids should take into consideration the presence of symptoms (often pain, bleeding, or infertility); the age and reproductive aspirations of the woman; and the number, size, and location of the fibroids. Most asymptomatic patients do not need specific treatment, requiring only periodic monitoring with imaging examinations^(22,23)^. Although the initial treatment for most patients with symptoms of abnormal bleeding is clinical, the definitive treatment for fibroids is surgical^(23)^. Typically, hysterectomy and myomectomy are the most effective treatments^(24)^. Alternatives to surgery include embolization of the uterine arteries and magnetic resonance imaging (MRI)-guided focused ultrasound ablation^(25)^. The key imaging aspects for the surgical treatment of fibroids are outlined in the following items.

### Uterine volume

**Figure 4:**
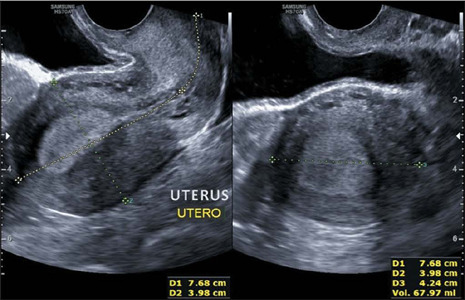
Transvaginal ultrasound image, in transverse and longitudinal views, showing the dimensions of the uterus.

It is recommended that the longitudinal, anteroposterior, and transverse diameters of the uterus be measured, because that provides the uterine volume in cm^3^, as shown in Figure 4, which is extremely useful in the surgical planning^(26,27)^. When the uterine volume exceeds 375 mL, the efficiency of transvaginal ultrasound in fibroid mapping is significantly lower than is that of MRI^(28)^.

### Number of fibroids

**Figure 5:**
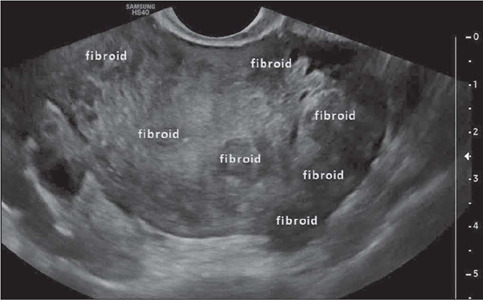
Transvaginal ultrasound image, in a cross-sectional view, showing myomatosis in a large uterus.

The number of fibroids will determine whether fibroid resection is feasible for symptom control. When there are numerous fibroids, radiologists should consider reporting a range of 10–20. Although it is not necessary to describe all lesions, a minimum number should be chosen^(27)^. Most previous studies have suggested that radiologists should describe no more than four non-submucosal fibroids and should describe all submucosal fibroids^(25,26,27)^, as depicted in Figure 5.

### Size

**Figure 6:**
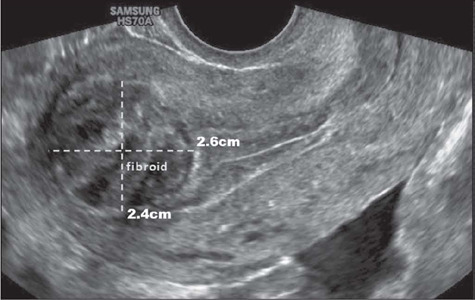
Transvaginal ultrasound image, in a longitudinal view, showing the dimensions of a fibroid.

It is recommended that each fibroid described in the report be systematically measured in three orthogonal planes, to obtain its volume in cm^3^, as illustrated in Figure 6. Knowledge of the size of each fibroid helps the gynecologist estimate the probability that the fibroids are (collectively) the direct cause of the symptoms and determine the best surgical approach in each case^(28)^.

### Location

**Figure 7:**
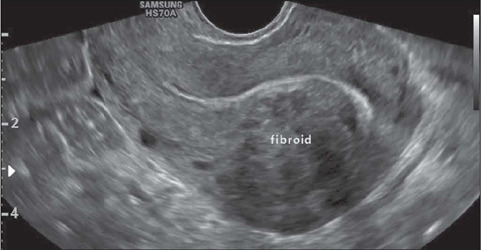
Transvaginal ultrasound image, in a longitudinal view, showing a submucosal (FIGO 2) fibroid in the anterior wall of a retroverted uterine body.

It is essential to register the location of each fibroid as being in the wall of the uterus—anterior, posterior, or lateral (right or left)—in the uterine fundus, or global (Figure 7). For example, when the fibroid is located in the lateral wall or in the uterine fundus, there is a greater degree of complexity in the hysteroscopic surgical procedure^(29)^.

### FIGO classification

**Figure 8:**
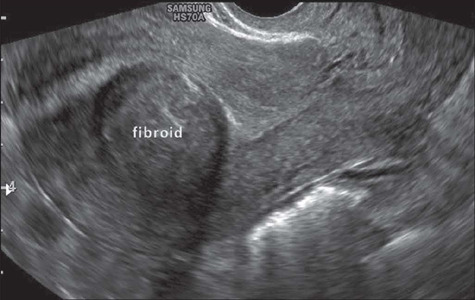
Transvaginal ultrasound image, in a longitudinal view, showing a submucosal (FIGO 2) fibroid with an intramural component > 50%.

Submucosal (FIGO 0, 1, and 2) uterine fibroids constitute a common cause of menorrhagia and dysmenor-rhea because they project into the endometrial cavity. For women who wish to become pregnant, submucosal fibroids are especially worrisome because they can cause infertility or miscarriage^(30)^. Therefore, such fibroids require surgical treatment, regardless of size. Treatment often includes hysteroscopic resection. For symptomatic patients who have no desire to become pregnant, hysterectomy can be an option. Hysteroscopic myomectomy of a bulky FIGO 2 fibroid, as depicted in Figure 8, can be difficult and might require a two-stage surgical procedure or uterine artery embolization^(31)^.

**Figure 9:**
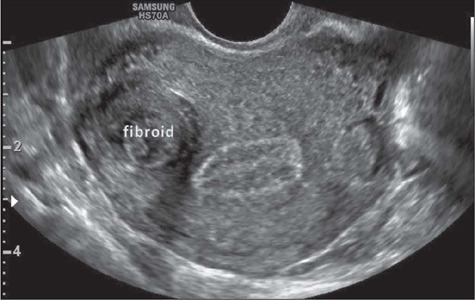
Transvaginal ultrasound image, in a cross-sectional view, showing an intramural (FIGO 4) fibroid.

Fibroids without a submucosal component (intramural and subserosal fibroids) that cause symptoms of mass effect in the uterine cavity or adjacent structures such as the bladder and bowel can be treated with embolization, myomectomy, or hysterectomy if there is no possibility of or desire for pregnancy. Accurately differentiating FIGO 2 fibroids from FIGO 3 and 4 fibroids is critical, because the surgical approach differs^(32)^: FIGO 2 fibroids are resected by hysteroscopy; and FIGO 3 and 4 fibroids are resected by video-assisted laparoscopy or laparotomy. Figure 9 shows an intramural FIGO 4 fibroid.

**Figure 10:**
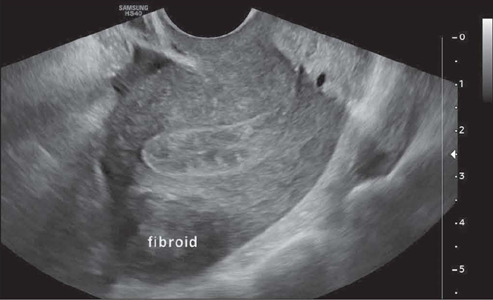
Transvaginal ultrasound image, in a longitudinal view, showing a subserosal fibroid with an intramural component < 50% (i.e., a FIGO 6 fibroid) in the posterior wall of the uterine fundus.

Treatment of bulky symptomatic fibroids and of bulky subserosal (FIGO 5, 6, and 7) fibroids in adjacent structures includes embolization, video-assisted laparoscopic myomectomy, and laparotomy. Due to their vascular pedicle, FIGO 7 fibroids are also at risk of twisting, shedding, or becoming parasitized in the pelvis. For FIGO 5, 6, and 7 fibroids, the treatment options include embolization, laparoscopic resection, laparotomy or hysterectomy^(33)^. Figure 10 shows a FIGO 6 fibroid in the uterine fundus.

**Figure 11:**
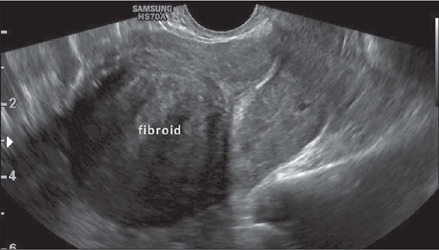
Transvaginal ultrasound image, in a longitudinal view, showing a hybrid (FIGO 2-5) fibroid in the uterine fundus.

A FIGO 2-5 fibroid, which is less than 50% submucosal and less than 50% subserosal (Figure 11), is a commonly found hybrid type of fibroid. Due to the size and extent of such a fibroid, treatment includes targeted therapy such as MRI-guided focused ultrasound or embolization, although hysterectomy can be required if the fibroid is extensive^(34,35)^.

### Myometrial mantle

**Figure 12:**
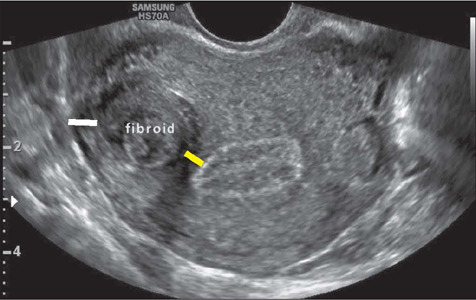
Transvaginal ultrasound image, in a cross-sectional view, showing an intramural (FIGO 4) fibroid, with the measurement of the outer mantle (distance from the serous surface, white line) and of the inner mantle (distance from the endometrial surface, yellow line).

The thickness of the myometrial mantle can be measured on transvaginal ultrasound (Figure 12). Various authors consider the outer myometrial mantle (distance from the fibroid margin to the serous surface) and the inner myometrial mantle (distance from the fibroid margin to the endometrial surface) to be key factors for hysteroscopic resection of submucosal fibroids. Some studies suggest that, in FIGO 2 fibroids, there is a greater chance of uterine rupture during resection if the outer myometrial mantle is smaller than 0.5 cm^(36)^.

### Adenomyosis

**Figure 13:**
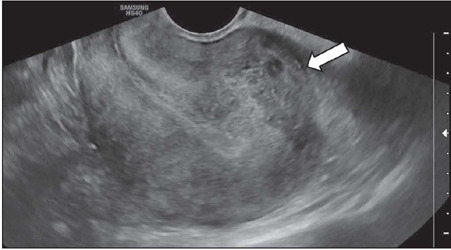
Transvaginal ultrasound image showing a retroverted uterus with adenomyosis infiltrating the posterior wall (arrow).

Recognition of adenomyosis is critical because it can change the treatment approach, patient counseling, and expectations. Adenomyosis, as shown in Figure 13, is defined as diffuse or focal invasion of the endometrial basal layer into the myometrium, can cause fibroid-like symptoms, and is identified on ultrasound as thickening or irregularity of the junctional zone, asymmetry of the myometrial walls, acoustic bands in the myometrium (myometrial stratification into fan-shaped shadowing), subendometrial/myometrial echogenic linear striations, myometrial cysts, and increased vascularization on Doppler, with penetrating vessels in the affected area^(37)^.

### Endometriosis

**Figure 14:**
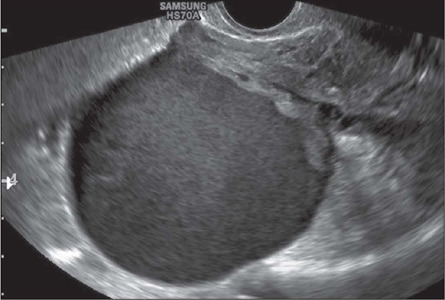
Transvaginal ultrasound image showing an ovarian endometrioma.

A preoperative diagnosis of endometriosis directly influences the planning of the surgical treatment of fibroids and the composition of the multidisciplinary surgical team. Therefore, screening for endometriosis on routine transvaginal ultrasound, based on the International Deep Endometriosis Analysis group consensus^(38)^, should be encouraged and should be performed with a practical, dynamic, four-step ultrasound approach: routine evaluation of the uterus and adnexa with special attention to ultrasound signs of adenomyosis and the presence or absence of endometriomas (Figure 14); evaluation of indirect soft markers, such as site-specific sensitivity and ovarian mobility; assessment of the pouch of Douglas status by realtime ultrasound testing for the “sliding sign”; and identification of deep infiltrating endometriotic nodules in the anterior and posterior compartments, which necessitates evaluation of the bladder, vaginal vault, retrocervical region, uterosacral ligaments, and bowel.

## SALINE INFUSION ULTRASOUND AND 3D ULTRASOUND FOR PREOPERATIVE EVALUATION OF FIBROIDS

Sonohysterography consists of transvaginal ultrasound combined with the infusion of sterile saline through a catheter into the uterine cavity. This minimally invasive 3D technique allows clear delineation of the uterine cavity. It is superior to two-dimensional ultrasound for the diagnosis of intrauterine abnormalities such as polyps and submucosal fibroids. In a pooled analysis using the gold standard (hysteroscopy) as the reference^(39)^, saline infusion ultrasound was found to have a sensitivity of 92% and a specificity of 90%, compared with 64% and 90%, respectively, for transvaginal ultrasound. Finally, 3D ultrasound can facilitate the spatial assessment, allowing more accurate characterization and localization of fibroids than what is achieved with two-dimensional ultrasound. Multiplanar views, especially the coronal view, have improved the description of fibroids on ultrasound^(40)^.

## PROPOSAL FOR A STRUCTURED ULTRASOUND REPORT TEMPLATE FOCUSING ON THE PREOPERATIVE EVALUATION OF PATIENTS WITH FIBROIDS

**Figure 15:**
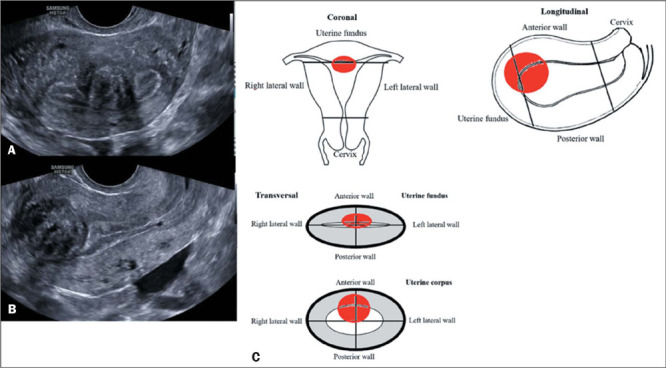
Transvaginal ultrasound, in cross-sectional and longitudinal views (A and B images, respectively), showing a uterine fibroid. Schematic drawings for reporting fibroids (C).

Although the FIGO classification system has provided gynecologists with a well-standardized framework for characterizing uterine fibroids, there is still significant variability across transvaginal ultrasound reports in terms of the quality of the descriptions of fibroids. Incomplete descriptions of fibroids or associated lesions such as adenomyosis and endometriosis can raise questions or lead to inappropriate surgical planning^(40)^. Consequently, a structured, illustrated model of an ultrasound report, standardizing the description of uterine fibroids—based on the critical criteria for surgical management, the FIGO classification of uterine fibroid location, and the MUSA group descriptors—could be useful for sonographers and physician examiners. A structured, accurately illustrated ultrasound report of fibroids allows gynecologists to choose the best treatment for the patient, be it hysteroscopy, laparoscopy, laparotomy, or embolization^(41,42)^. The proposed report template is shown in the Appendix. In addition, bowel preparation can be added if specifically requested by the attending physician. Another relevant topic when considering the imaging evaluation of patients with fibroids is illustrating the imaging findings with drawings or sketches (Figure 15), which is also strongly recommended and valued by surgeons and patients because it provides a roadmap for treatment^(43,44,45)^.

## CONCLUSION

There are key points in the characterization of fibroids that help gynecologists plan the surgical treatment and have the potential to allow complications and treatment failure to be avoided. The structured, illustrated ultrasound report model proposed here, which is based on those critical points, could improve patient counseling and treatment planning, as well as facilitating the selection of the most appropriate medical or surgical treatment strategy.

## Figures and Tables

**Figure:**
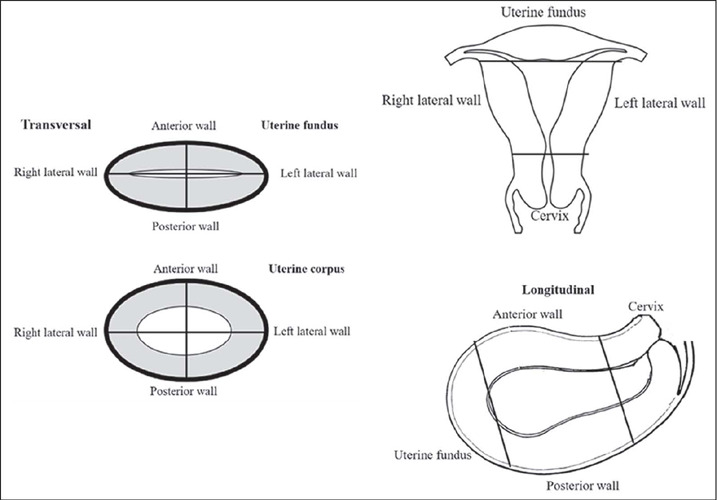
Schematic drawings for reporting fi broids

## Appendix Proposed template for reporting uterine fibroids on preoperative ultrasound examinations.

Reporting fibroids on ultrasound for surgical planning • INDICATION FOR THE EXAMINATION

– Asymptomatic patient ( )
– Evaluation of a clinical finding
  Pelvic pain ( )
  Menorrhagia ( )
  Infertility ( )
– Fibroid follow-up ( )
– Follow-up after surgical fibroid treatment ( )

• TECHNIQUE Examination performed with a device (model/manufacturer) with convex (abdominal) and intracavitary (transvaginal) transducers and with/without bowel preparation. • FINDINGS
**Middle pelvic compartment**
*– Uterus:* in (*anteversion/retroversion*) position, with regular outer contours, myometrium with preserved echotexture, except in the areas of myometrial nodules and normal mobility (positive sliding sign). *Uterine biometry:* __ **×** __ **×** __ cm (volume: __ cm^3^). Note the presence of solid, hypoechoic, and heterogeneous nodules, with regular contours and well-defined limits, consistent with fibroids. The table below shows the main aspects:

**Table:**

Fibroid	FIGO classification	Dimensions (cm)	Localization	Inner mantle	Outer mantle
1					
2					
3					
4					
5					
6					

– *Endometrium: centered/displaced*, of uniform echogenicity, *trilaminar/ echogenic* pattern, measuring __ mm thick, junctional zone (*regular/irregular*) – *Right ovary:* parauterine, with normal contours, normal echotexture, and normal mobility, measuring __ **×** __ **×** __ cm (volume: __ cm^3^) – *Left ovary:* parauterine, with normal contours, normal echotexture, and normal mobility, measuring __ **×** __ **×** __ cm (volume: __ cm^3^) Report of painful sensitivity on mobilization with a transducer Yes ( ) No ( )
**Anterior pelvic compartment**
*Bladder:* good repletion; thin, regular walls; and homogeneous anechoic content. There was no evidence of endometriotic lesions in the bladder. In the search for adhesions, there was mobility and anatomical sliding of the bladder wall against the anterior wall of the uterus (positive sliding sign).
**Posterior pelvic compartment**
There is no evidence of endometriotic foci in the retrocervical region and uterosacral ligaments. There are no evident signs of thickening or nodules in the intestinal loops or rectum detectable without bowel preparation. Signs of adenomyosis

( ) Absent
( ) Focal
( ) Diffuse

• CONCLUSIONS

– Myometrial nodule(s) compatible with fibroid(s), type 0/1/2/3/4/5/6/7/8 (FIGO classification)
– Number of fibroids: __
– Number of fibroids with submucosal component: __
– Number of fibroids without submucosal component: __
– Mass effect on the endometrial cavity: ( ) Yes ( ) No
– Presence of submucosal fibroid in the uterine fundus: ( ) Yes ( ) No
– Presence of submucosal fibroid in the lateral wall: ( ) Yes ( ) No
– Focal/diffuse adenomyosis
– Ovaries with normal ultrasound findings
– Ovarian reserve: Normal ( ) Low ( )
– Endometrioma in the right/left ovary
– Adhesive processes in the vesicouterine pouch/rectouterine pouch
– Anterior pelvic compartment with deep endometriosis; endometriosis mapping with bowel preparation recommended
– Posterior pelvic compartment with deep endometriosis; endometriosis mapping with bowel preparation recommended
Schematic drawings for reporting fibroids
